# A Collection of Pins

**Published:** 1869-11

**Authors:** 


A collection of twenty-five pins, very well made, has just been placed in the Louvre, Paris. They were found in the subterranean vaults of Thebes, and were made more than three thousand years ago, showing that the modern invention is only a reinvention.

